# Correlation between the small dense LDL level and nonalcoholic fatty liver disease: Possibility of a new biomarker

**DOI:** 10.1097/MD.0000000000021162

**Published:** 2020-07-10

**Authors:** Ha Won Hwang, Jung Hwan Yu, Young-Joo Jin, Young Ju Suh, Jin-Woo Lee

**Affiliations:** a Department of Internal Medicine, Inha University Hospital, Inha University School of Medicine; b Department of Biomedical Sciences, College of Medicine, Inha University, Incheon, South Korea.

**Keywords:** biomarker, nonalcoholic fatty liver disease, small dense low-density lipoprotein

## Abstract

Small dense low-density lipoprotein (sdLDL) is a distinct low-density lipoprotein (LDL) cholesterol subclass that has been reported to be associated with metabolic disease. On the other hand, the relationship between the sdLDL level and the nonalcoholic fatty liver disease (NAFLD) severity is unclear. In this study, the sdLDL level was measured in patients with NAFLD to assess its potential as a biomarker for evaluating NAFLD. One hundred and twenty-six patients diagnosed with NAFLD at a single referral hospital from January 2018 to August 2019 were enrolled. The lipoprotein profile was analyzed from a blood test of NAFLD patients, and transient elastography (TE, Fibroscan) was performed to evaluate the degree of NAFLD. Among the 126 patients, 83 patients that could confirm the lipoprotein profile and TE results were finally enrolled in the study. The controlled attenuation parameter (CAP) value obtained from TE did not show any correlation with the total cholesterol, LDL. But, the sdLDL level showed a significant positive correlation with the CAP value (*r* = 0.237, *P* = .031), and the sdLDL/LDL ratio also showed a significant positive correlation with the CAP value (*r* = 0.235, *P* = .032). The liver stiffness (LS) measured by TE and the sdLDL level were positively correlated in patients with NAFLD (rho = 0.217, *P* = .049). The sdLDL/LDL ratio also showed a significant positive correlation with the LS value (rho = 0.228, *P* = .038). In addition, the fatty liver index also showed a significant positive correlation with the sdLDL/LDL ratio (*r* = 0.448, *P* = .000). In this study, the sdLDL level measured by a blood test of NAFLD patients showed a positive correlation with the CAP value and LS, which indicate the degree of hepatic steatosis and fibrosis. These results suggest the possibility of the sdLDL level as a new biomarker of NAFLD, but further studies will be needed to support these results.

## Abstract 2

Supplemental Digital Content is available in the text

## 1 Introduction

Nonalcoholic fatty liver disease (NAFLD) is the most common chronic liver disease in the world, with a global prevalence of approximately 25%.^[1]^ Moreover, the prevalence of NAFLD has increased gradually with the increase in obesity and has become a significant health care issue. NAFLD is defined as the presence of ≥5% of hepatic steatosis. NAFLD can be categorized histologically into nonalcoholic fatty liver (NAFL) and nonalcoholic steatohepatitis (NASH). NAFL is defined as the presence of at least 5% hepatic steatosis without evidence of hepatocellular injury in the form of hepatocyte ballooning. NASH is defined as the presence of at least 5% hepatic steatosis and inflammation with hepatocyte injury (e.g., ballooning), with or without fibrosis.^[2]^ NAFLD is considered to be the hepatic manifestation of metabolic syndrome.^[3,4]^ Many studies have shown that the prevalence of metabolic syndrome in NAFLD patients is high.^[5]^ NAFLD is also commonly accompanied by metabolic diseases, such as obesity, diabetes mellitus, dyslipidemia, and polycystic ovary syndrome.^[6,7]^ NAFLD is a progressive disease. It can progress to nonalcoholic steatohepatitis (NASH) and fibrosis, and ultimately to cirrhosis and hepatocellular carcinoma (HCC). Therefore, it is important to assess the severity of NAFLD accurately and provide the appropriate treatment for the condition of the disease.

Dyslipidemia is one of the common comorbid conditions found in patients with NAFLD, and is characterized by the increased levels of triglycerides (TG) and low-density lipoprotein (LDL) and decreased levels of high-density lipoprotein (HDL).^[8,9]^ Dyslipidemia in NAFLD has several other important features, one of which is the increased small dense low-density lipoprotein (sdLDL) particles, which is a subtype of LDL.^[10–12]^ sdLDL is a distinct LDL cholesterol subclass that is associated with metabolic disease. Most studies on sdLDL focused on the risk of cardiovascular disease and the development of atherosclerosis, but recent studies have reported an increase in the number of sdLDL particles in patients with NAFLD.^[10–12]^ In addition, some studies have shown that NAFLD patients with steatohepatitis or fibrosis have higher sdLDL levels than those with NAFL.^[13,14]^ For example, Sonmez et al compared 17 NAFL patients with 24 NASH patients, and showed that sdLDL increased in NASH patients compared to NAFL patients.^[14]^ Based on these results, it is believed that the sdLDL levels are associated with the severity of NAFLD. On the other hand, few studies have examined the correlation between the sdLDL levels and NAFLD severity.

Until now, a liver biopsy is regarded as the golden standard to evaluate the degree of NAFLD by establishing the presence of steatohepatitis and fibrosis.^[15]^ On the other hand, its use is limited to routine clinical practice because of its high cost and potential complications, and the measurement range is also limited. Therefore, many noninvasive biomarkers and radiological modalities have been proposed to diagnose the severity of NAFLD, but there are few suitable methods that can be used in general clinical practice. Therefore, this study examined the clinical significance of the sdLDL level in patients with NAFLD to assess its potential as a noninvasive biomarker of NAFLD.

## 2 Material and methods

### 2.1 Study subjects

Patients diagnosed with NAFLD were recruited from a gastroenterology outpatient clinic of Inha University Hospital (Incheon, South Korea) from January 2018 to August 2019. The inclusion criteria were adults over 18 years of age, patients diagnosed with NAFLD, and patients who voluntarily agreed to this study and signed written consent. NAFLD was defined by imaging tests demonstrating fatty liver disease without significant alcohol consumption (20 g/day and 30 g/day for women and men, respectively), the use of drugs that cause fatty liver, and liver disease caused by other causes, such as the viruses. Patients were excluded if they have liver diseases other than NAFLD (e.g., viral hepatitis, toxic hepatitis, autoimmune hepatitis, etc.), malignant tumors, including HCC, underlying diseases that may affect the evaluation of fatty liver (e.g., severe kidney disease, severe lung disease, severe cardiovascular disease, etc.), and immune diseases. Patients who received treatment that could affect the liver function test within 1 month prior to the study and patients who took medications that could cause fatty liver disease within three months prior to the study were also excluded. At first, 126 patients were enrolled. Among these patients, 24 patients who did not undergo TE, 12 patients who did not undergo lipid profile analysis, and seven patients whose written consent was not confirmed were excluded from the study. Finally, 83 patients were included in the study.

The patients were checked for height, weight, body mass index (BMI), drug and alcohol history, and other medical history through history taking and a physical examination. The complete blood counts, liver function tests, and biochemistry analyses (total cholesterol, triglycerides, HDL, LDL, total protein, albumin, fasting glucose, fasting insulin, etc.) were performed. In addition, lipoprotein profile tests were conducted. The study was approved by the Institutional Review Board of Inha University Hospital, Incheon, South Korea (Approval number: INHAUH 2019-05-033-002).

### 2.2 Lipoprotein profile

Twelve distinct lipoprotein subclasses were assessed, including very low-density lipoproteins (VLDLs), three intermediate-density lipoproteins (IDL-A, IDL-B, IDL-C), seven LDLs (LDL-1, LDL-2, LDL-3, LDL-4, LDL-5, LDL-6, LDL-7), and HDL. LDL can be classified into seven subfractions, from LDL-1 to LDL-7. Of the seven subfractions, LDL-1 and LDL-2 are large and buoyant LDLs, and LDL-3 to LDL-7 correspond to sdLDL. The purpose of this study was to examine the relationship between the NAFLD severity and the sdLDL level; hence, the sdLDL level and the sdLDL/LDL ratio were analyzed.

Different laboratory procedures can be used to separate LDL subfractions. In addition, the results of the LDL subfractions may differ according to which method is used. Ultracentrifugation and electrophoresis are used mostly to determine the LDL subfractions. On the other hand, a gold standard method for assessing the LDL subfractions has not been established yet.^[16]^ This study analyzed the LDL subfractions using the following method. The LDL subfraction was analyzed using 3% polyacrylamide gel tube electrophoresis (Lipoprint TM LDL System; Quantimetrix, Redondo Beach, CA, USA) according to a previous procedure.^[17]^ Electrophoretic mobility (Rf) was calculated qualitatively and quantitatively using the Lipoprint LDL system Template and the Lipoware software (property of Quantimetrix; Redondo Beach, CA), respectively. Rf of the LDL subfractions was estimated using the Rf between the very low-density lipoprotein (VLDL) fraction (Rf 0.0) and the HDL fraction (Rf 1.0). LDL is distributed as seven bands, with Rfs of 0.32, 0.38, 0.45, 0.51, 0.56, 0.60, and 0.64 corresponding to LDL subclasses 1 to 7, respectively. LDL subclasses 3 to 7 were defined as small dense LDL subfractions. Prior to the study, blood tests were performed on 254 healthy individuals to examine the normal range of sdLDL levels using this immunoturbidimetric assay. Of the blood samples obtained from these volunteers, only those samples (n = 125) that met the NECP guidelines for a desirable lipid status were analyzed. The expected normal values, which are defined as the 95% confidence interval (mean ± 2SD) for the sdLDL level, were calculated to be 0 to 6.3 mg/dL. Therefore, subgroup analysis was performed by dividing the NAFLD patients into a group with a sdLDL level above 6.3 mg/dL and a group below 6.3 mg/dL. The characteristics of the normal sdLDL group were compared with the increased sdLDL group.

### 2.3 NAFLD severity

The NAFLD severity was evaluated by transient elastography (TE, Fibroscan) as a noninvasive evaluation method. Liver fibrosis and steatosis were assessed by the liver stiffness (LS) and controlled attenuation parameter (CAP) score measured by TE. In addition to this, the fatty liver index (FLI) was also used to evaluate the NAFLD severity. FLI is the widely used index for the diagnosis of hepatic steatosis. It is based on routine measurements in clinical practice such as BMI, waist circumference, TG, and gamma-glutamyl-transferase, and thus, it is easy to employ. And it had an accuracy of 0.84 (95% CI 0.81 to 0.87) in detecting fatty liver.^[18]^ The correlation between the NAFLD severity and the sdLDL level was investigated by evaluating the correlation between the sdLDL level measured by blood tests and the LS, CAP, and FLI.

### 2.4 Statistical analysis

Statistical analyses were performed using SPSS software. The categorical variables were analyzed using the Pearson Chi-square test. For the continuous variable analysis, a *t* test and a Mann-Whitney test were used as a parametric and nonparametric method, respectively. The Pearson correlation method and Spearman correlation method were used as a parametric and nonparametric method to determine if the sdLDL level was associated with the NAFLD severity. A *P* value < .05 was considered significant.

## 3 Results

One hundred and twenty six patients were enrolled from January 2018 to August 2019. Among these patients, 83 patients, who underwent lipid profile analysis, liver imaging tests, and TE were included in the study. Table 1 lists the clinical and laboratory characteristics and the TE results of the patients. Twenty-three patients (27.7%) were also diagnosed with diabetes mellitus (DM). The average BMI of the patients was 29.54, indicating that most were overweight. The mean white blood cell (WBC) of the patients was 7044 /μL, and the mean hemoglobin (Hb) 15.1 g/dL, mean platelet 258317 /μL, mean total bilirubin 0.71 mg/dL, mean aspartate aminotransferase (AST) 53.01 IU/L, mean alanine aminotransferase (ALT) 93.24 IU/L. A blood test was performed to check the lipoprotein profile of the patients. Table 2 lists the lipid profile characteristics of the patients. The mean total cholesterol level of the patients was 197.6 mg/dL, and the mean TG 177.1 mg/dL, mean HDL 42.9 mg/dL, mean LDL 113.9 mg/dL, mean VLDL 40.55 mg/dL, mean IDL-A 12.78 mg/dL, mean IDL-B 9.07 mg/dL, mean IDL-C 21.57 mg/dL. The mean sdLDL level of the patients was 10.6 mg/dL and mean sdLDL/LDL ratio was 0.09.

**Table 1:**
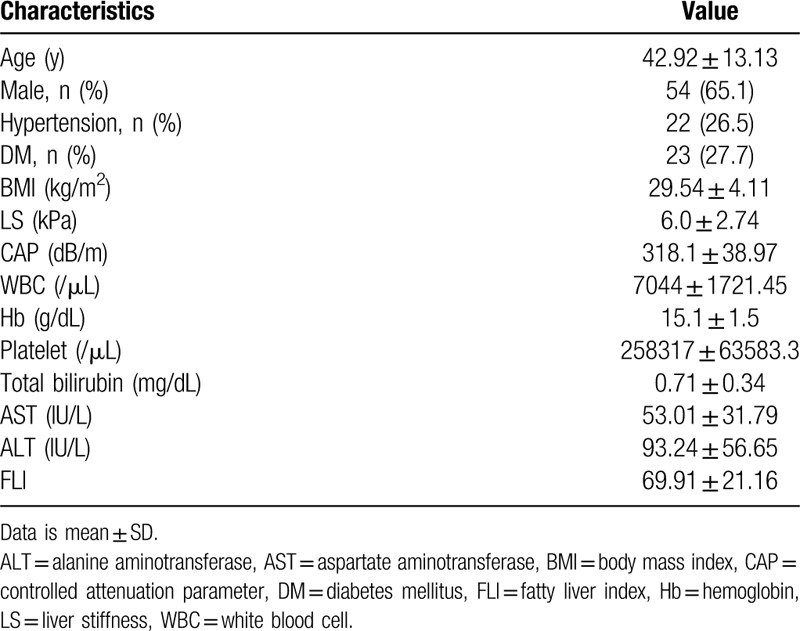
Clinical and laboratory characteristics of the subjects (n = 83).

**Table 2:**
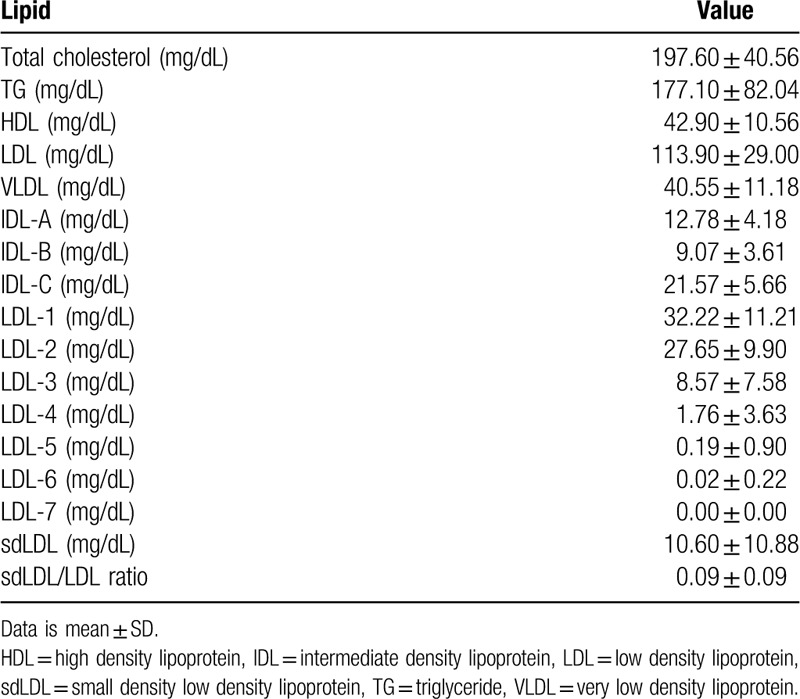
Lipid profile of the subjects (n = 83).

To evaluate the NAFLD severity, TE was performed, and the FLI was calculated. Table 3 lists the results of NAFLD severity analysis. The LS and CAP cut off values of TE are not consistent across the world and vary according to country or institution. In the author's institution, the LS and CAP cut off values were determined with reference to articles.^[19,20]^ In this institution, the LS cut off value for fibrosis F2 in the NAFD patients was 7, and 8.7 for fibrosis F3, 10.3 for fibrosis F4. The cut off value for steatosis grade S2 (>34% of hepatic steatosis) in the NAFLD patients was 258, and 283 for steatosis grade S3 (>67% of hepatic steatosis). When analyzing the CAP and LS results based on these criteria, there were 67 patients with fibrosis F0 or F1, six patients with F2, seven patients with F3, and three patients with F4. Only 10 patients were considered to have advanced fibrosis. In addition, steatosis grade S2 was noted in 11 patients and S3 in 68 patients. Most patients had grade S2 or S3 steatosis.

**Table 3:**
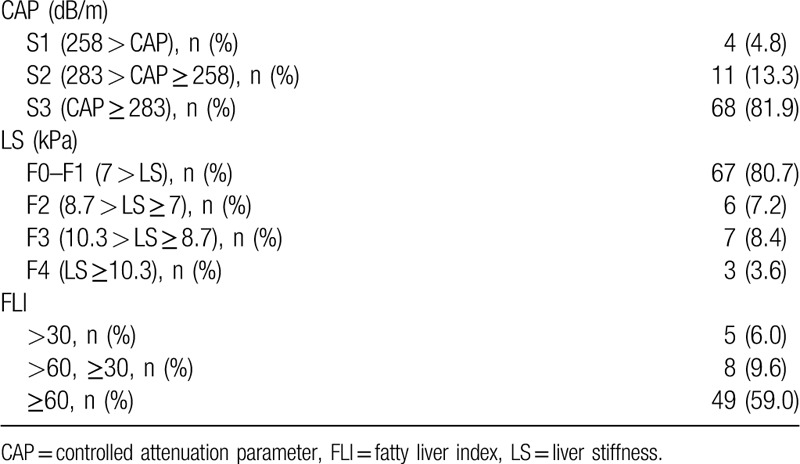
NAFLD severity.

BMI, waist circumference, GGT, TG are required for FLI calculation. However, there were some patients whose waist circumference were not measured, so their FLI were not calculated. So, there was a difference between the total number of patients and the number of patients with FLI calculated. FLI was calculated in 62 of a total of 83 patients. In a study on FLI by Bedogni et al, a FLI < 30 can be used to rule out (sensitivity = 87%; negative likelihood ratio = 0.2) and a FLI ≥ 60 can be used to rule in hepatic steatosis (specificity = 86%; positive likelihood ratio = 4.3).^[18]^ When calculating the FLI, five patients were below 30 and 49 patients were above 60.

**Figure 1:**
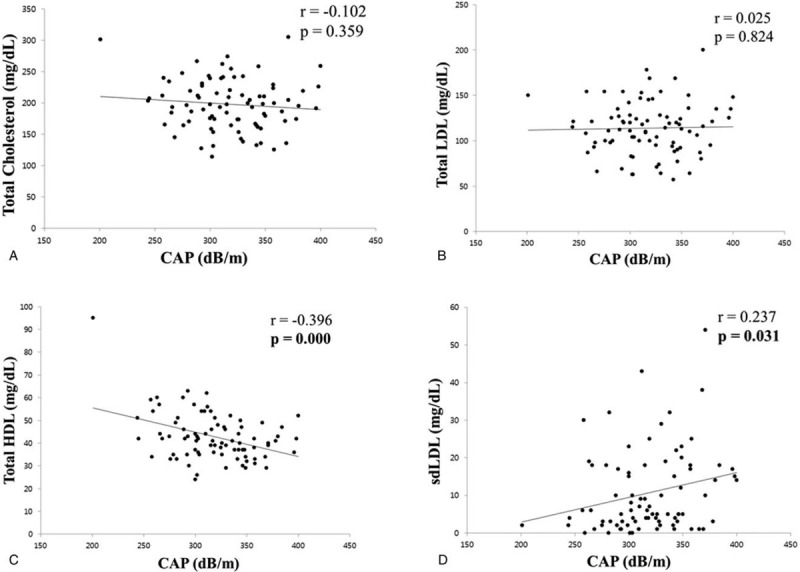
Correlation between lipid profile and CAP. (A) Correlation between total cholesterol and CAP. (B) Correlation between total LDL and CAP. (C) Correlation between total HDL and CAP. (D) Correlation between sdLDL and CAP. CAP = controlled attenuation parameter, HDL = high density lipoprotein, LDL = low density lipoprotein, sdLDL = small dense low density lipoprotein.

**Figure 2:**
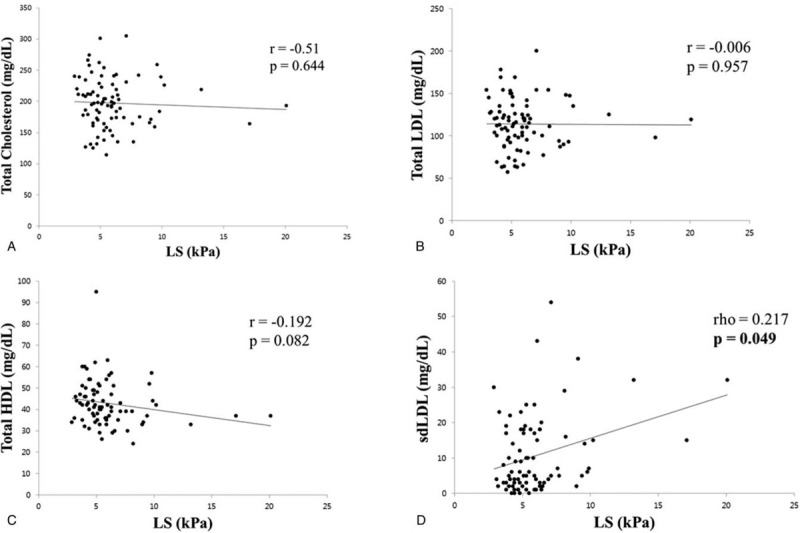
Correlation between lipid profile and LS. (A) Correlation between total cholesterol and LS. (B) Correlation between total LDL and LS. (C) Correlation between total HDL and LS. (D) Correlation between sdLDL and LS. HDL = high density lipoprotein, LDL = low density lipoprotein, LS = liver stiffness, sdLDL = small dense low density lipoprotein.

The correlation between the NAFLD severity and lipoprotein profile was analyzed. First, the correlation between the lipoprotein profile and the steatosis severity was analyzed using the CAP obtained with TE (Fig. 1). The total cholesterol and total LDL level did not show a significant correlation with the CAP. On the other hand, the total HDL level showed a significant negative correlation with the CAP (*r* = −0.396, *P* = .000) and the sdLDL level had a significant positive correlation with the CAP (*r* = 0.0237, *P* = .031). The correlation between the lipoprotein profile and fibrosis severity was analyzed using the LS obtained by TE. The total cholesterol, total LDL, and HDL did not show a significant correlation with the LS, but the sdLDL level showed a significant positive correlation with the LS (rho = 0.217, *P* = .049) (Fig. 2).

**Figure 3:**
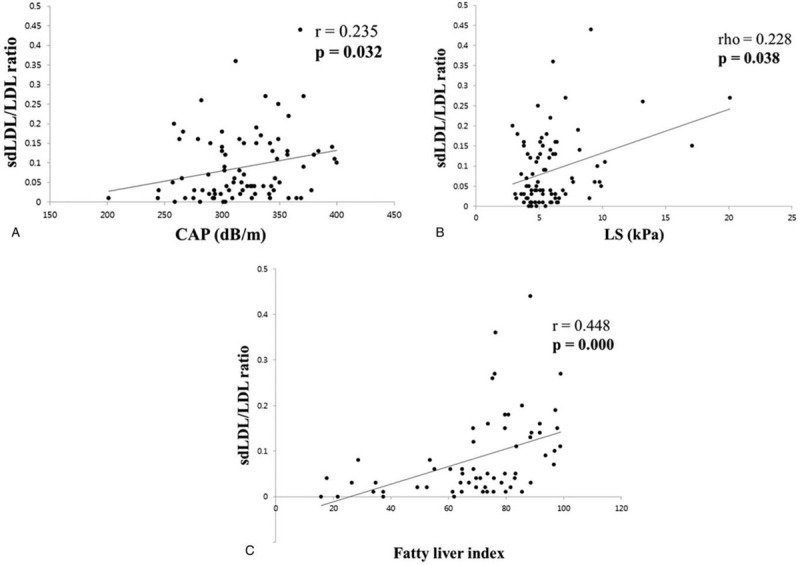
Correlation between sdLDL/LDL ratio and NAFLD severity. (A) Correlation between sdLDL/LDL ratio and CAP. (B) Correlation between sdLDL/LDL ratio and LS. (C) Correlation between sdLDL/LDL ratio and FLI. CAP = controlled attenuation parameter, FLI = fatty liver index, LDL = low density lipoprotein, NAFLD = nonalcoholic fatty liver disease, sdLDL = small dense low density lipoprotein.

To analyze the correlation between the sdLDL/LDL ratio and NAFLD severity, the correlations between sdLDL/LDL and CAP, LS, FLI were analyzed (Fig. 3). The sdLDL/LDL ratio showed a significant positive correlation with the CAP and LS (*r* = 0.235, *P* = .032 and rho = 0.228, *P* = .038, respectively). The sdLDL/LDL ratio also showed a significant positive correlation with FLI (*r* = 0.448, *P* = .000). We further analyzed the correlations between the level of sdLDL, the sdLDL/LDL ratio, and the subgroups of NAFLD (LS, CAP, and FLI). The level of sdLDL and the sdLDL/LDL ratio both showed significantly positive correlations with the LS and FLI subgroups, but not with the CAP subgroups (Supplemental Table 1). Subgroup analysis was performed by dividing the NAFLD patients into two groups: those with an sdLDL level above 6.3 mg/dL and below 6.3 mg/dL (Table 4). In the comparison of the characteristics of the group with a normal sdLDL level and the group with a high sdLDL level, the CAP and FLI were significantly different between the two groups. (*P* = .015 and *P* = .000, respectively).

**Table 4:**
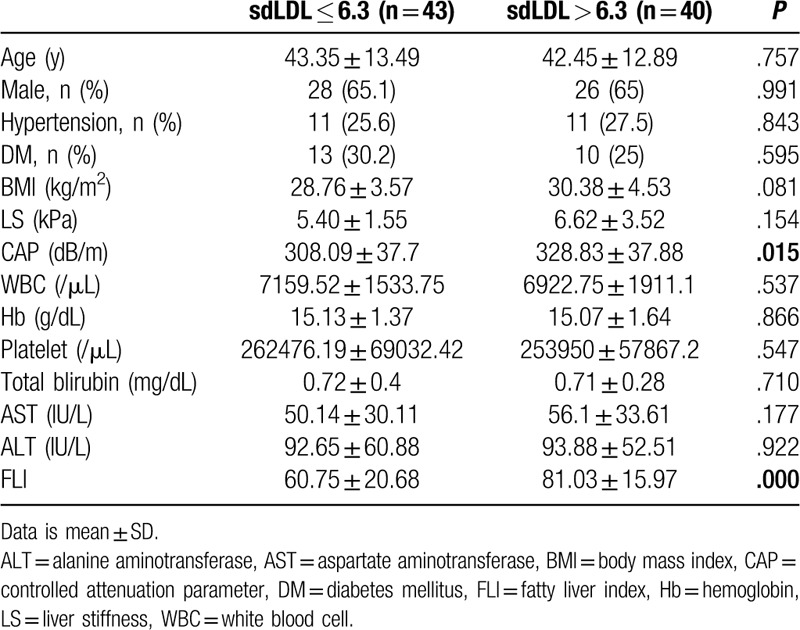
Subgroup analysis classified by sdLDL 6.3.

## 4 Discussion

In this study, the sdLDL level measured in patients with NAFLD showed a significant positive correlation with the CAP and LS values, which indicate the degree of hepatic steatosis and fibrosis, respectively. The sdLDL/LDL ratio also showed a meaningful correlation with the degree of NAFLD, similar to sdLDL. Furthermore, the sdLDL/LDL ratio showed positive correlations with the FLI which is the index for the diagnosis of hepatic steatosis. Previous studies have shown that the sdLDL level increases in NAFLD patients.^[10–12]^ To the best of the authors’ knowledge, however, few studies have analyzed the correlation between the sdLDL level and the NAFLD severity. Therefore, this study is meaningful in that it investigated the correlation between the sdLDL level and NAFLD severity and confirmed the positive correlation in NAFLD patients. These results also suggest that the sdLDL level could be used as a biomarker for assessing the steatosis and fibrosis severity in NAFLD patients.

NAFLD has a range of processes ranging from NAFL to cirrhosis and shows a difference in prognosis as the disease progresses. The degree of hepatic steatosis in NAFLD patient is known to predict the prevalence of metabolic disease such as diabetes,^[21,22]^ and the degree of hepatic fibrosis can predict the mortality in patients with NAFLD.^[23]^ On the other hand, the best diagnostic methods for hepatic steatosis and advanced fibrosis have not been fully established, and many diagnostic methods have been studied. To date, a liver biopsy is regarded as a gold standard for assessing the level of fibrosis and severity in NAFLD patients, but a liver biopsy does not reflect the characteristics of the entire liver; the results may differ according to the examined site. The cost and potential complications are also limitations of this test.^[15]^ Therefore, many noninvasive tools for the diagnosis of fibrosis have been developed, including clinical decision aids, serum biomarkers, or imaging.^[24]^

Many types of medical equipment, such as liver ultrasonography, liver vessels Doppler ultrasonography, magnetic resonance imaging (MRI), and TE have been studied as noninvasive tools for evaluating NAFLD patients.^[2,25]^ Magnetic resonance imaging proton density fat fraction (MRI-PDFF) is an excellent tool for identifying and quantifying the degrees of steatosis in patients with NAFLD.^[26]^ In addition, MRI-PDFF shows excellent accuracy compared to ultrasonography and TE in obese patients. On the other hand, MRI-PDFF is a relatively expensive technique for noninvasive measurements of steatosis, and it is not available for all institutions. Ultrasonography is the most widely used imaging test for evaluating NAFLD, but the test is operator dependent. And it is not reliable for evaluating hepatic fibrosis. TE is also a widely used set of medical equipment for NAFLD patients with fairly high accuracy.^[27,28]^ Although TE is less accurate than MRI and biopsy, it is a relatively quick, inexpensive method and it is clinically more accessible compared to these tests. Therefore, we used TE as an examination method for evaluating hepatic steatosis and fibrosis.

To evaluate the severity of NAFLD, many noninvasive fibrosis scores (e.g., NAFLD fibrosis score, FIB-4 index, AST to platelet ratio index, etc) using the clinical parameters and biochemical measurements have been used.^[24]^ Although these scoring indicators may not play an absolute role in assessing NAFLD, they play a supporting role along with imaging tests for evaluating the degree of NAFLD. The serum biomarkers for the diagnosis of NAFLD, including the enhanced liver fibrosis (ELF) panel, fibrotest, etc., have been also studied.^[29,30]^ These methods, however, are not used widely in the clinical field. In this study, the sdLDL level showed a positive correlation with the severity of fibrosis. Therefore, the authors suggest that the sdLDL level can be used to generate more accurate noninvasive fibrosis scores by using them together with other clinical parameters and biochemical measurements (e.g., age, BMI, platelet count, AST, ALT, etc), rather than a single marker for fibrosis; further studies will be needed. sdLDL is a distinct subclass of LDL which has the most atherogenic properties^[16]^ and it is elevated in atherosclerotic disorders, such as cardiovascular disease (CVD).^[31]^ Some studies have also shown that the sdLDL level is associated with the severity of CVD.^[32]^ In NAFLD patients, CVD is one of the important comorbidities, and one of the major causes of death associated with NAFLD.^[23,33,34]^ Although this study focused on the relationship between the sdLDL level and the severity of NAFLD, it is thought that the sdLDL level can also be associated with the CVD severity in NAFLD patients. Briefly, the sdLDL level has important implications on the complications of atherosclerotic diseases, including CVD in NAFLD patients, in addition to the NAFLD severity. However, there has been little research on this issue. From this point of view, further attention and research on the sdLDL level in NAFLD patients are needed.

This study had some limitations. First, it was a single center study, which may not reflect the situation worldwide. Therefore, a multicenter with a larger number of subjects is needed. The sdLDL level and NAFLD severity showed a significant but weak positive correlation in this study, which is probably because it was a single-center study with a small number of subjects. A positive correlation between the sdLDL level and the NAFLD severity may be more apparent if a multicenter with a larger number of subjects is performed. Second, a liver biopsy was not performed to evaluate the degree of steatosis and fibrosis in NAFLD patients. Although a liver biopsy is the gold standard for evaluating hepatic fibrosis in NAFLD patients, this institution does not perform liver biopsies on NAFLD patients routinely due to the cost, possible complications, and patient rejection. Instead, TE was used to evaluate the severity of NAFLD. Although TE is not a complete replacement for a liver biopsy, it has high sensitivity and specificity for an evaluation of NAFLD, and it shows a significant correlation with the pathology results.^[27,28]^ In addition, to complement TE, the FLI was used as the index for the diagnosis of hepatic steatosis. However, considering the accuracy of various information provided by the liver biopsy, it is thought that the results of this study can be confirmed more clearly if a study in which a liver biopsy is added in the future is conducted.

In conclusion, the sdLDL level and the sdLDL/LDL ratio have a positive correlation with the severity of NAFLD measured by TE, unlike other lipoproteins. Furthermore, the FLI, which is the index for the diagnosis of hepatic steatosis, was found to have a significant positive correlation with the sdLDL/LDL ratio. Based on these results, the sdLDL level could be used as a new noninvasive tool to evaluate the severity of NAFLD steatosis and fibrosis, but further studies will be needed.

## Author contributions

HW Hwang, JH Yu, and JW Lee were responsible for the concept and design of the study, as well as the acquisition, analysis, and interpretation of the data and the drafting of the manuscript. YJ Suh helped with the statistical analysis and interpretation of the data. YJ Jin helped with the interpretation of the data.

## Supplementary Material

Supplemental Digital Content
