# Supplementary material for: Correlation between the small dense LDL level and nonalcoholic fatty liver disease: Possibility of a new biomarker
Source: Medicine (Baltimore). 2020 Jul 10;99(28):e21162. doi: 10.1097/MD.0000000000021162 (PMC7360208; doi:10.1097/MD.0000000000021162)
Supplement: Supplemental Digital Content [file medi-99-e21162-s001.docx]

| Supplemental table 1. Correlation between sdLDL and NAFLD subgroups | | | |
| --- | --- | --- | --- |
|  | | sdLDL | sdLDL/LDL ratio |
| LS subgroups | rho | 0.287 | 0.291 |
|  | p value | **0.008** | **0.008** |
| CAP subgroups | rho | 0.097 | 0.118 |
|  | p value | 0.383 | 0.288 |
| FLI subgroups | rho | 0.415 | 0.370 |
|  | p value | **0.001** | **0.003** |
| CAP, controlled attenuation parameter; LS, liver stiffness; FLI, fatty liver index. | | | |
